# Effects of organic fertilizer proportion on the distribution of soil aggregates and their associated organic carbon in a field mulched with gravel

**DOI:** 10.1038/s41598-022-15110-z

**Published:** 2022-07-07

**Authors:** Shaoping Du, Zhongming Ma, Juan Chen, Liang Xue, Chaonan Tang, Tawheed M. E. Shareef, Kadambot H. M. Siddique

**Affiliations:** 1 Institute of Vegetables, Gansu Academy of Agricultural Sciences, Lanzhou, 730070 Gansu China; 2 Gansu Academy of Agricultural Sciences, Lanzhou, 730070 Gansu China; 3 Institute of Economic Crops and Beer Materials, Gansu Academy of Agricultural Sciences, Lanzhou, 730070 Gansu China; 4 Institute of Soil, Fertilizer and Water-Saving Agriculture, Gansu Academy of Agricultural Sciences, Lanzhou, 730070 Gansu China; 5 Faculty of Agriculture Dept. of Agric. Engineering, University of Khartoum, Shambat, Sudan; 6 The UWA Institute of Agriculture, The University of Western Australia, Perth, WA 6001 Australia

**Keywords:** Environmental sciences, Agroecology

## Abstract

Gravel and sand mulching is an indigenous technology that has been used for increasing soil temperature and improving crop yield and water use efficiency for at least 300 years in northwestern China. However, long-term application of inorganic fertilizer with gravel and sand mulch could decrease the soil organic carbon content, and how to improve soil fertility under gravel and sand mulching remains largely unknown. Thus, we evaluated the effects of the application of inorganic (chemical) and organic (manure) fertilizers on the distribution of soil aggregates and their associated organic carbon in a field mulched with gravel and sand. A 5-year (2014–2018) field experiment was conducted in the arid region of northwestern China. Total organic carbon (TOC), permanganate oxidizable carbon (POC), TOC reserves in soil aggregates with different particle sizes, and watermelon (*Citrullus lanatus*) productivity in gravel-mulched fields were analysed for the following six fertilization modes: no N fertilizer input as a control (CK), N fertilizer without organic fertilizer (CF), and organic fertilizer replacing 25%, 50%, 75%, and 100% of mineral nitrogen (recorded as OF-25%, OF-50%, OF-75% and OF-100%, respectively). The results showed that, higher manure to nitrogen fertilizer ratios were positively correlated with the percentage of soil macroaggregates (> 0.25 mm), mean weight diameter (MWD), TOC and POC concentrations, and their ratios in different particle sizes. Compared with CF, the treatments with 50% to 100% organic fertilizer significantly increased TOC storage (5.91–7.84%) in the soil profile (0–20 cm). Moreover, the CF treatment did not increase SOC concentrations or TOC storage, compared with CK. The fruit yield (2014–2018) of watermelon significantly increased by an average of 31.38% to 45.70% in the treatments with 50% to 100% organic fertilizer, respectively, compared with CF. Our results suggest that the partial replacement of chemical fertilizer with organic manure (OF-50%, OF-75% and OF-100%) could increase the proportion of macroaggregates, POC and TOC concentrations, and TOC stock in aggregates with different particle size and improve the yield of watermelon in the gravel fields of arid northwestern China mulched with gravel and sand.

## Introduction

Gravel and sand mulching has been used for at least 300 years in the arid area of northwestern China^1,2^. A layer of gravel and sand approximately 10 cm thick lies on the soil surface. Numerous studies have shown that gravel and sand mulching can reduce erosion from water and wind^3^, limit the accumulation of salt near the soil surface^4^, increase soil temperature, extend the whole growing season^5^, and retain moisture by increasing water infiltration and reducing evaporative water loss compared to bare soil surfaces^6^. Overall, the gravel and sand mulching facilitates microhydrological management and promotes increased crop biomass and crop yield ^7^. Given these beneficial aspects of gravel and sand mulching, it is unsurprising that its application is widely used in agriculture in arid and semiarid environments, such as in the United Arab Emirates^8^, New Mexico^9^, and Lanzorate of the Canary Islands^10^. At present, there are approximately 100,000 ha of gravel and sand-mulched fields in China, of which about 75% are used for growing watermelon (*Citrullus lanatus*) ^1^, which has become a highly economically lucrative crop in the arid northwest.

**Figure 1:**
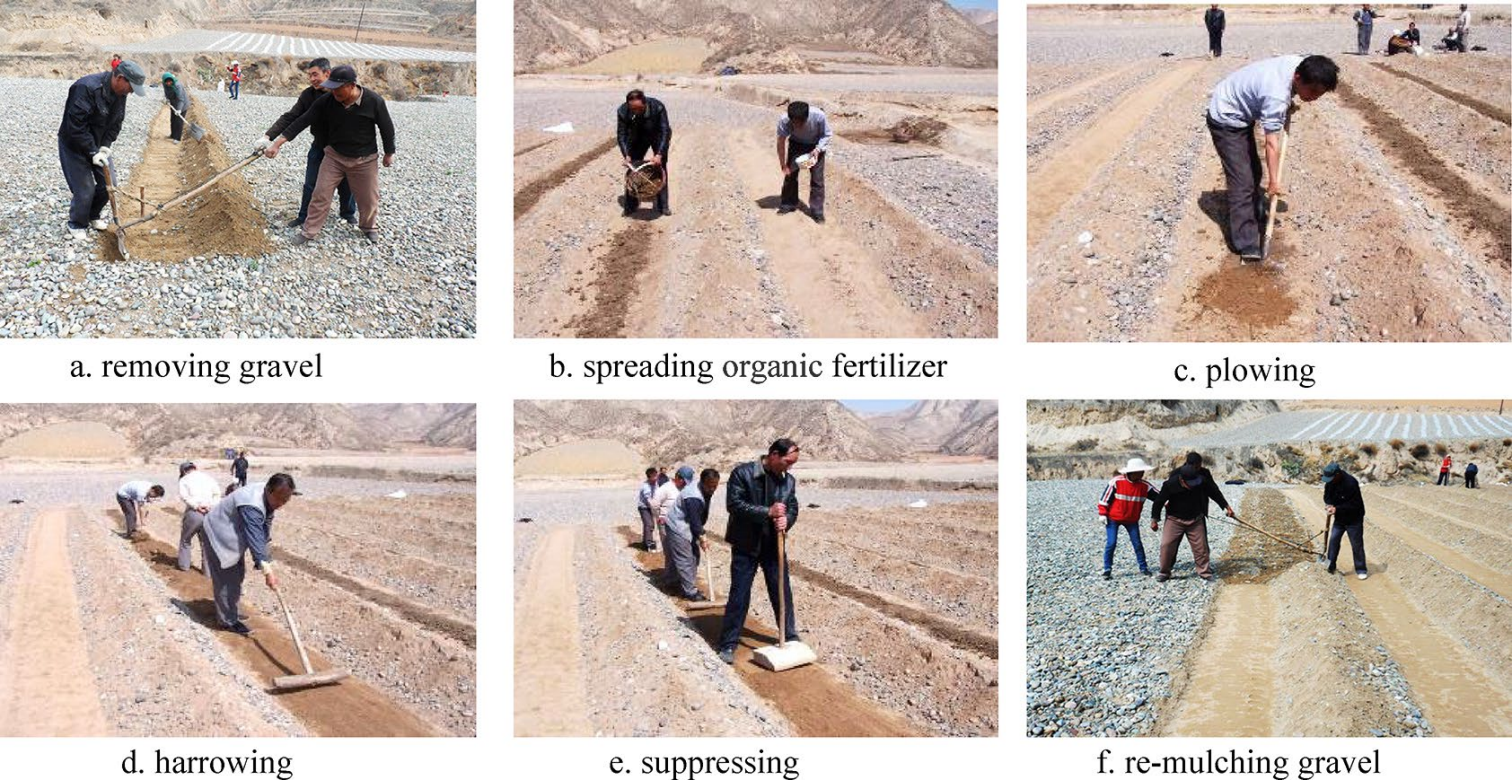
Application process of farm manure in a gravel-mulched field. *Note*: There were not any authors of the manuscript in this figure. All the people involved are farmers temporarily employed in the field experiment, and the written informed consent was obtained from these farmers for publication.

Despite the many benefits of utilizing gravel and sand mulching, it may also lead to soil deterioration. The addition of organic fertilizer would be a good method to improve soil fertilizer. However, the application of organic fertilizer, especially farmyard manure, involves many complex procedures due to the lack of special fertilization equipment, including removing the existing gravel mulch, spreading organic fertilizer, plowing, restoring soil surface uniformity, and re-mulching with gravel (Fig. 1). In China, this process is often accomplished via manual labour. Therefore, farmers largely prefer to apply chemical and inorganic fertilizers rather than organic manure to minimize the costs involved^1^. Unfortunately, the long-term application of inorganic fertilizer with gravel sand mulch could reduce the macroaggregate content as well as lower the mean weight diameter (MWD)^11^. Thus, gravel-mulched fields often show decreased Soil organic carbon (SOC) with continuous cropping^2,11^ and, consequently, degradation of other physical, chemical, and microbial properties^12–14^. In China, soil degradation in gravel-mulched fields in the arid northwestern region has significantly negative impacts on the watermelon crop.

Soil organic carbon (SOC) serves as an important sink for many nutrients, drives the soil nutrient cycle, helps to maintain structural stability, aids in air and water infiltration, promotes water retention, and reduces erosion^15^. It is an important index reflecting soil quality and soil health, which directly affects soil fertility and crop yield^16,17^. Lal^17^ found that the soil organic carbon pool increased by 1 mg ha^−1^ y^−1^ increasing food grain yield by 32 million mg y^−1^ in developing countries. Studies have found that SOC is mainly distributed in macroaggregates (> 1 mm), and its content increases with aggregate diameter^18,19^. The microaggregates (< 0.25 mm) can effectively reduce the loss of SOC, and accumulation of greater SOC in microaggregates effectively promotes macroaggregates^20,21^. Meanwhile, SOC accumulation improves soil aggregation, yielding an even greater capacity for SOC storage^22^. Thus, this positive interaction between SOC and aggregate formation achieved a physical protection of SOC. In recent years, numerous studies have found nutrient management is an important factor governing SOC and aggregates in soils^23–31^. Moreover, the application of organic materials or combining organic fertilizer and inorganic fertilizer have significantly positive effect in increasing the SOC content, improving macroaggregates formation and enhancing the stability of soil aggregates^23,24,26–29,31^.

However, few prior studies have been conducted to investigate the potential of replacing chemical fertilizer with manure in soil restoration and maintenance in gravel-mulched watermelon fields within the sandy, arid region of northwestern China. Therefore, in this 5-year study, we partially replaced chemical fertilizer with manure in a 20-year-old gravel-mulched watermelon field in northwestern China with the objectives of evaluating the effects on fruit yield of watermelon, sizes and abundance of soil aggregates, and relative distribution of SOC and SOC stocks retained by the different aggregate sizes. We expect that our results have certain guiding significance for soil quality improvement and the optimization of watermelon nutrient management strategies in gravel-mulched fields in northwestern China.

## Materials and methods

### Study area

**Figure 2:**
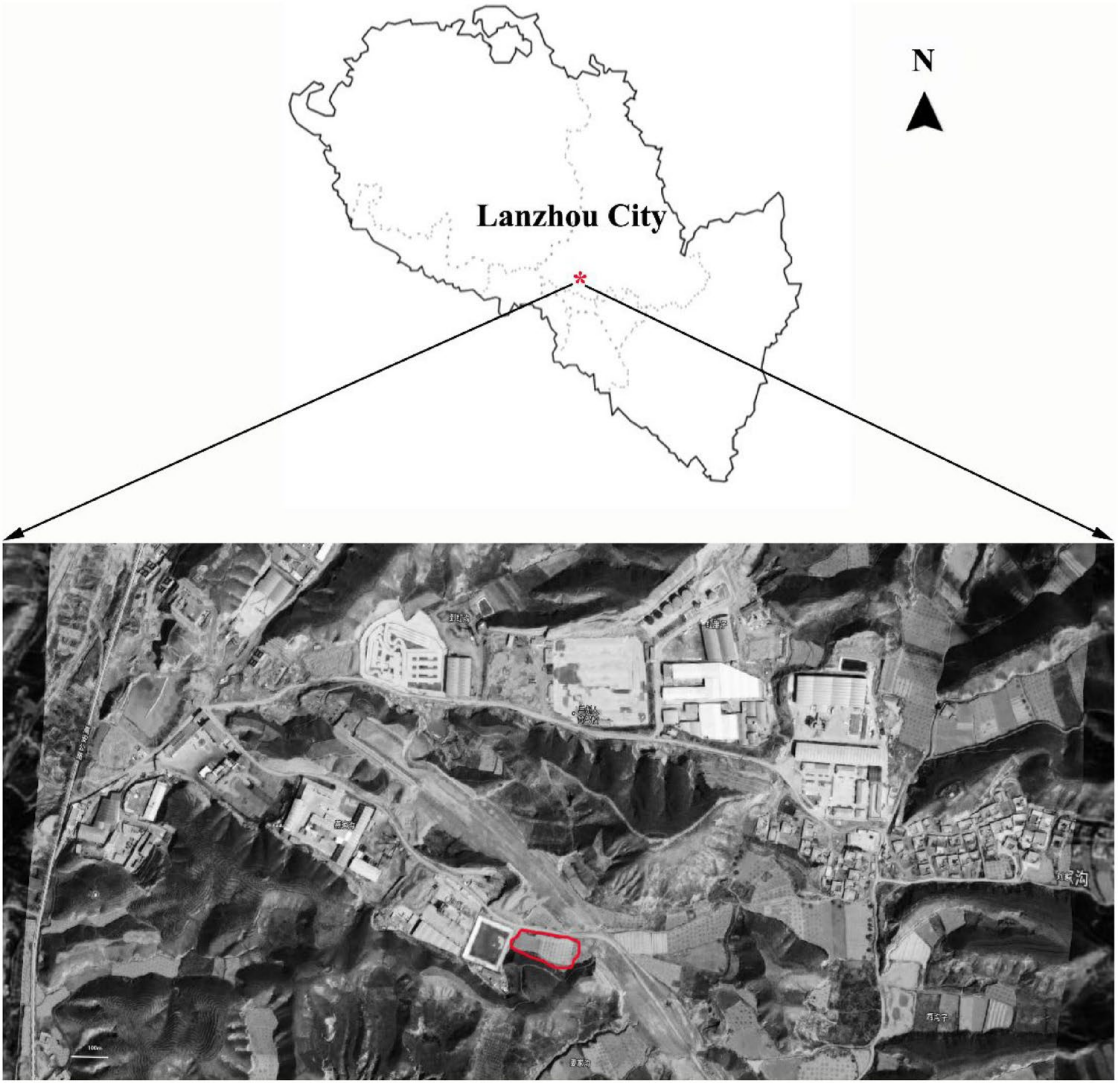
Location of the study area (Lanzhou, China).

This 5-year study (2014–2018) was conducted on at experimental site established by the Gansu Academy of Agricultural Sciences (GAAS) Gaolan Research Station of Ecology and Agriculture (36°l3ʹN, 103°42ʹE), Gaolan County, Lanzhou city, Gansu Province, China. The site has an elevation of 1830 m above sea level (Fig. 2). The mean annual precipitation is 260 mm, of which 60% falls between July and September. The mean annual temperature is 7.0 °C, monthly maximum temperatures range from 20.7 °C in July to − 9.1 °C in January, and the frost-free period is approximately 140–150 days long.

The soil at the experimental site is a haplicorthicaridisols and silt loam, and the depth of the soil cover is 1 m. The gravel mulch was distributed on the soil surface to a depth of 8.5 cm and comprise 85% gravel and 15% soil by volume. Additional basic properties of the soil at the experimental site that were observed in 2014 are presented in Table 1.

**Table 1:** Basic chemical and physical properties of the soil (0–20 cm) in 2014 at the experimental field site.

pH	Total nitrogen (g kg^–1^)	Organic matter (g kg^–1^)	Avail. phosphorus (mg kg^–1^)	Avail. potassium (mg kg^–1^)	Bulk density (Mg m^–3^)	Sand (g kg^–1^)	Silt (g kg^–1^)	Clay (g kg^–1^)	Soil type
8.72	0.31	2.59	13.02	66.42	1.48	123	669	208	Silt loam

### Experimental design

The watermelon (*Citrullus lanatus*) cultivar, 'Xi Sha Rui Bao', was used as the experimental material. The inorganic fertilizer used in this experiment included urea (N, 46%), calcium superphosphate (P_2_O_5_, 12%) and potassium sulfate (K_2_O, 52%). The organic fertilizer used in this experiment was cow manure, and its nutrient content was determined by conventional chemical analysis methods (organic matter 311 = g kg^−1^, total nitrogen = 15 g kg^−1^, total phosphorus = 12 g kg^−1^, and total potassium = 19 g kg^−1^)^32^.

The experiment was arranged in a completely randomized design with three replications of six fertilization treatments:

1. No nitrogen fertilizer (CK, NPK dosage: 0 kg ha^−1^, 170 kg ha^−1^ and 260 kg ha^−1^, supplied by inorganic fertilizers).
2. Inorganic fertilizer (CF, NPK dosage: 200 kg ha^−1^, 170 kg ha^−1^ and 260 kg ha^−1^, supplied by inorganic fertilizers) applied in accordance with the optimized amount of fertilizer selected from the preliminary work of the project.
3. Organic fertilizer replacing 25% of mineral nitrogen (OF-25%, NPK dosage: 200 kg ha^−1^, 170 kg ha^−1^ and 260 kg ha^−1^): 75% of the total N-fertilizer was supplied by inorganic fertilizer, and 25% was supplied by cow manure.
4. Organic fertilizer replacing 50% of mineral nitrogen (OF-50%, NPK dosage: 200 kg ha^−1^, 170 kg ha^−1^ and 260 kg ha^−1^): 50% of the total N-fertilizer was supplied by inorganic fertilizer, and 50% was supplied by cow manure.
5. Organic fertilizer replacing 75% of mineral nitrogen (OF-75%, NPK dosage: 200 kg ha^−1^, 170 kg ha^−1^ and 260 kg ha^−1^): 25% of the total N-fertilizer was supplied by inorganic fertilizer, and 75% was supplied by cow manure.
6. Organic fertilizer replacing 100% of mineral nitrogen (OF-100%, NPK dosage: 200 kg ha^−1^, 170 kg ha^−1^ and 260 kg ha^−1^): all of the total N-fertilizer was supplied by cow manure.

The NPK fertilizer amounts of OF-25%, OF-50%, OF-75% and OF-100%, were calculated according to the replacement amount of inorganic nitrogen fertilizer and the nutrient content of cow manure based on the equal fertilization amount. The specific application amount is shown in Table 2.

**Table 2:** The fertilizer ratio and amount for the different treatments in 2014–2018.

Treatments		N (kg ha^−1^)								P (kg ha^−1^)						K (kg ha^−1^)						
		Inorganic fertilizer				Organic fertilizer (Cow manure)/Basal fertilizer		Total N-fertilizer		Inorganic fertilizer		Organic fertilizer (Cow manure)		Total P-fertilizer/Basal fertilizer		Inorganic fertilizer				Organic fertilizer (Cow manure)/Basal fertilizer	Total K-fertilizer	
		Basal fertilizer		Top dressing												Basal fertilizer		Top dressing				
CK		–		–		–		–		170		–		170		104		156		–	260	
CF		60		140		–		200		170		–		170		104		156		–	260	
OF-25%		45		105		50 (3333.33)		200		130		40 (3333.33)		170		78.67		118		63.33 (3333.33)	260	
OF-50%		30		70		100 (6666.67)		200		90		80 (6666.67)		170		53.33		80		126.67 (6666.67)	260	
OF-75%		15		35		150 (10,000)		200		50		120 (10,000)		170		28		42		190 (10,000)	260	
OF-100%		–		–		200 (13,333.33)		200		10		160 (13,333.33)		170		2.67		4		253.33 (13,333.33)	260	

The plot size was 10 m × 6.4 m = 64 m^2^.

Each year during the course of the study (2014–2018), watermelon seeds of ‘Xi Sha Rui Bao’ were planted in the middle of April, and harvested in the middle of July. The row spacing was 1.0 m, the distance between holes was 0.6 m, and one seed was planted in each hole, representing a total of 11,335 plants per hectare. Before sowing, all of the organic fertilizer (cow manure), 30% of the total inorganic N-fertilizer, all of the inorganic P-fertilizer, and 40% of the total inorganic K-fertilizer were applied as basal fertilizer at a depth of 20 cm to the planting row of watermelon according to the steps shown in Fig. 1. The remaining 70% of the total inorganic N-fertilizer and 60% of the total inorganic K-fertilizer were applied as top dressing (spread on fields) at the watermelon vine extension stage and fruit expansion period, respectively (Table 2). There was no irrigation in any of the treatments, so the watermelon relied exclusively on precipitation and soil moisture.

### Soil sampling

In 2018, after the final watermelon harvest, we collected 3 soil samples under the gravel mulching layer in each planting row. The soil samples consisting of 10 cm × 10 cm in sections at a depth of 20 cm were randomly collected using a spade. Then we transported the samples in hard plastic boxes from the field to the laboratory to preserve the original physical structure of the soil. In the lab, the collected fresh soil samples were air-dried at 4 °C according to the method of Dorodnikov et al. (2009) so that the soil moisture of all samples reached 10–12%^33^. Then, these air-dried samples were divided into two subsamples: one subsample was used to analyse soil aggregates and their associated organic carbon, and one subsample was used as a backup.

### Soil analysis

The stability of the air-dried soil aggregates was determined according to the method of Kemper and Rosenau (1986)^34^. The air-dried soil sample was broken into soil particles < 5 mm along the natural textures. These 100 g soil particles were placed on the uppermost layer of a series of sieves (from high to low apertures: 2 mm, 1 mm, 0.25 mm) in an automatic sieve shaker (Retsch AS 200, Germany). After 2 min, aggregates of > 2 mm, 1–2 mm, 0.25–1 mm and < 0.25 mm were achieved under the condition of 1.5 cm amplitude and 30 times/min frequency. Then, the mass of the agglomerates with the corresponding size class was obtained by weighing. We used the resulting dry mass from the sieves to compute the mean weight diameter (MWD) as

1 $$\text{MWD} = \sum {\mathrm{X}}_{\mathrm{i}}{\mathrm{W}}_{\mathrm{i}}$$

where X_i_ is the mean diameter of aggregates in a sieve fraction (i) in mm, and W_i_ is the proportion of the total sample mass represented by the sieve fraction.

For the aggregate fractions from each sieve, we also analysed total organic carbon (TOC) concentrations using the dichromate oxidation method with external heating^35^. Moreover, to measure permanganate oxidizable carbon (POC) concentrations in the aggregate fractions, we used the permanganate oxidation method with oxidation by 333 mmol L^−1^ KMnO_4_ measured with a spectrophotometer at 565 nm^36^.

The contribution rate of aggregates to organic carbon (CAOC) was calculated according to the formula of Li et al. (2014)^37^.

2 $${\text{CAOC}}\,(\%)=\frac{{\mathrm{A}}_{\mathrm{i}}\times {\mathrm{C}}_{\mathrm{i}}}{\mathrm{TOC}}\times 100$$

where A_i_ is the percentage represented by a sieved fraction (i) and C_i_ is the percentage organic carbon content represented by the fraction.

We determined the TOC storage (Mg ha^−1^) in different aggregate fractions using the following equation^38^:

3 $$\text{TOC storage}=\mathrm{TOC\,concentration}\times \mathrm{D}\times \mathrm{BD}\times 0.1$$

where D is the thickness of the soil layer (cm) and BD is the bulk density (Mg m^−3^).

### Watermelon yield

We harvested watermelon from each 64 m^2^ plot in the middle of July, which is a typical harvest time for this late-maturing watermelon cultivar. After harvesting watermelon fruit, we removed most of the aboveground biomass, including leaves and stems. For each year (2014–2018), we calculated yield results as kilograms of fruit biomass per plot and extrapolated them to ha, expressed as kg per ha.

### Statistical analyses

Data were statistically analysed using analysis of variance (ANOVA). The significance of the treatment effects was analysed using SPSS 17.0. Variance analysis and mean values (n = 3) were compared with the least significant difference (LSD) at the 5% level.

**Guideline statement:** The watermelon cultivar ‘Xi Sha Rui Bao’ is not endangered species, and the cultivation and collection of plant material, field studies and laboratory experiments are all in compliance with relevant national regulations and legal regulations.

## Results

### Aggregate size distribution

The proportion of large macroaggregates (≥ 1 mm) significantly decreased with decreasing particle size, but small aggregates (< 1 mm) increased with particle size (Table 3). The different fertilization treatments affected the aggregate size distribution, with the percentage of aggregates ≥ 1 mm increasing with organic manure application (Table 3). Replacing 75% or 100% inorganic-N with organic fertilizer (OF-75%, OF-100%) increased the percentage of aggregates in the > 5 mm, 2–5 mm, and 1–2 mm fractions by 18.73–29.63%, 28.28–38.22%, and 38.00–48.20%, respectively, relative to the CF treatment. In contrast, the percentage of aggregates < 1 mm decreased with organic manure application. In particular, the OF-75% and OF-100% treatments showed decreased percentages of aggregates in the 0.25–1 mm and < 0.25 mm fractions by 13.15–14.81% and 22.45–34.87%, respectively, relative to the CF treatment. Thus, at the 0–20 cm soil depth, aggregates ≥ 1 mm were sensitive to the addition of organic material in long-term gravel-mulched fields. The percentage of macroaggregates (> 0.25 mm) (R_0.25_) and MWD increased by 8.26–18.20% and 14.67–33.33% in the OF-50%, OF-75%, and OF-100% treatments, respectively, relative to CF. Thus, increased application rates of organic manure were correlated with an increase in macroaggregates and MWD.

**Table 3:** Size distribution (%) of water-stable aggregates in different fertilization treatments in a gravel-mulched field.

Treatments	Aggregate size (mm)					R_0.25_ (%)	MWD (mm)
	> 5	2–5	1–2	0.25–1	< 0.25		
CK	17.74 ± 1.34dC	13.88 ± 1.66cD	9.34 ± 0.45cE	23.43 ± 1.24abB	35.62 ± 1.37aA	64.39 ± 1.37d	1.46 ± 0.08d
CF	18.53 ± 0.90cdC	14.18 ± 0.83cD	9.42 ± 0.66cE	23.57 ± 0.48aB	34.30 ± 1.88aA	65.70 ± 1.88d	1.50 ± 0.05d
OF-25%	19.64 ± 1.19bcdB	14.84 ± 1.24cC	10.88 ± 0.82bcD	22.32 ± 1.42abB	32.32 ± 2.89abA	67.68 ± 2.89 cd	1.57 ± 0.06 cd
OF-50%	20.76 ± 1.88bcB	16.32 ± 1.66bcC	12.73 ± 1.76abD	21.05 ± 1.88abB	28.87 ± 1.74bcA	71.13 ± 1.74bc	1.72 ± 0.10bc
OF-75%	22.00 ± 1.60abB	18.19 ± 1.97abB	13.00 ± 1.30abC	20.47 ± 2.17bB	26.60 ± 3.66cdA	73.40 ± 3.66b	1.84 ± 0.16 ab
OF-100%	24.02 ± 1.56aA	19.60 ± 0.80aC	13.96 ± 1.33aD	20.08 ± 1.96bBC	22.34 ± 0.91dAB	77.66 ± 0.91a	2.00 ± 0.04a

Lower-case letters in the same column indicate significant differences in fertilization. Upper-case letters in the same row indicate significant differences in aggregate size (*p* < 0.05). CK, no N fertilizer; CF, N fertilizer without organic fertilizer; OF-25%, OF-50%, OF-75%, and OF-100%, 25%, 50%, 75%, and 100% organic fertilizer. R_0.25_, the percentage of macroaggregates (> 0.25 mm).

### TOC concentrations in aggregate fractions with different sizes

TOC concentrations increased with decreasing particle size, with the greatest TOC levels in the < 0.25 mm fraction (Table 4). The TOC concentrations within all aggregate sizes increased with higher rates of organic manure application. Specifically, relative to the CF treatment, TOC concentrations increased by 11.88–20.46%, 7.49–13.47%, 7.58–13.12% and 6.94–15.14% within macroaggregates (> 5 mm, 2–5 mm, 1–2 mm, 0.25–1 mm), respectively, and 9.22–16.48% within microaggregates (< 0.25 mm) in the OF-50%, OF-75%, and OF-100% treatments. No significant differences were observed in the TOC concentration between CF and CK treatments.

**Table 4:** Total organic carbon concentration (g per kg) within soil aggregates in different fertilization treatments in a gravel-mulched field.

Treatments	Aggregate size (mm)				
	> 5	2–5	1–2	0.25–1	< 0.25
CK	2.95 ± 0.21cC	3.23 ± 0.06 dB	3.31 ± 0.12dAB	3.16 ± 0.06cB	3.48 ± 0.04dA
CF	3.03 ± 0.30bcC	3.34 ± 0.10cdAB	3.43 ± 0.14cdAB	3.17 ± 0.06cBC	3.58 ± 0.05cdA
OF-25%	3.10 ± 0.17bcC	3.41 ± 0.10cB	3.58 ± 0.03bcAB	3.21 ± 0.10cC	3.71 ± 0.04cA
OF-50%	3.39 ± 0.13abCD	3.59 ± 0.08bBC	3.69 ± 0.16abB	3.39 ± 0.06bD	3.91 ± 0.08bA
OF-75%	3.49 ± 0.29aB	3.66 ± 0.05abAB	3.83 ± 0.08aA	3.51 ± 0.13abB	3.92 ± 0.11bA
OF-100%	3.65 ± 0.07aC	3.79 ± 0.14aBC	3.88 ± 0.02aB	3.65 ± 0.11aC	4.17 ± 0.17aA

Lower-case letters in the same column indicate significant differences in fertilization. Upper-case letters in the same row indicate significant differences in aggregate size (*p* < 0.05). CK, no N fertilizer; CF, N fertilizer without organic fertilizer; OF-25%, OF-50%, OF-75%, and OF-100%, 25%, 50%, 75%, and 100% organic fertilizer.

### POC concentration in different-sized aggregate fractions

The POC concentration followed a similar trend to that of TOC; namely, it increased in different-sized aggregate fractions with the increasing application rate of organic manure. We observed the greatest level of POC in the macroaggregate fraction of 0.25–1 mm (Table 5). The ranges of POC increased by 20.69–32.76%, 13.79–32.76%, 19.23–32.69%, 17.74–29.03%, and 14.29–26.79% in the > 5 mm, 2–5 mm, 1–2 mm, 0.25–1 mm, and < 0.25 mm aggregate fractions, respectively, relative to the CF treatment across the OF-50%, OF-75%, and OF-100% treatments. Overall, organic fertilization increased the POC concentration more within macroaggregates (> 0.25 mm) than micro-aggregates (< 0.25 mm).

**Table 5:** POC concentration (g per kg) within soil aggregates in different fertilization treatments in a gravel-mulched field.

Treatments	Aggregate size (mm)				
	> 5	2–5	1–2	0.25–1	< 0.25
CK	0.55 ± 0.08dAB	0.55 ± 0.03cAB	0.50 ± 0.06cB	0.61 ± 0.03cA	0.55 ± 0.05dAB
CF	0.58 ± 0.06cdA	0.58 ± 0.06cA	0.52 ± 0.08bcA	0.62 ± 0.09cA	0.56 ± 0.05cdA
OF-25%	0.63 ± 0.09bcdAB	0.61 ± 0.04bcAB	0.59 ± 0.02abcB	0.69 ± 0.04bcA	0.62 ± 0.01bcAB
OF-50%	0.70 ± 0.08abcAB	0.66 ± 0.05bAB	0.62 ± 0.05abB	0.73 ± 0.04abA	0.64 ± 0.02bAB
OF-75%	0.73 ± 0.04abAB	0.76 ± 0.04aA	0.66 ± 0.10aB	0.77 ± 0.04abA	0.66 ± 0.03abB
OF-100%	0.77 ± 0.10aAB	0.77 ± 0.04aAB	0.69 ± 0.05aB	0.80 ± 0.01aA	0.71 ± 0.01aB

Lower-case letters in the same column indicate significant differences in fertilization. Upper-case letters in the same row indicate significant differences in aggregate size (*p* < 0.05). CK, no N fertilizer; CF, N fertilizer without organic fertilizer; OF-25%, OF-50%, OF-75%, and OF-100%, 25%, 50%, 75%, and 100% organic fertilizer.

### Percentage of POC to TOC

The percentage of POC concentration to TOC content decreased with decreasing particle size; that is, the percentage was higher within macroaggregates (> 0.25 mm) than microaggregates (< 0.25 mm) with the highest values in the 0.25–1 mm aggregate fraction (Table 6). The percentage of POC to TOC within some macroaggregates (1–2 mm,0.25–1 mm) was significantly higher in the OF-50%, OF-75%, and OF-100% treatments, than in the CF treatment, but no significant differences occurred within microaggregates (< 0.25 mm). Overall, the percentage of POC to TOC increased with increasing application rates of organic manure in different-sized aggregate fractions.

**Table 6:** Percentage of TOC represented by POC in different fertilization treatments in a gravel-mulched field.

Treatments	Aggregate size (mm)				
	> 5	2–5	1–2	0.25–1	< 0.25
CK	18.51 ± 0.81cAB	17.02 ± 0.33cBC	15.22 ± 0.32cD	19.32 ± 0.44bA	15.64 ± 1.61aCD
CF	19.12 ± 0.32bcAB	17.32 ± 0.70bcBC	15.14 ± 0.62cD	19.44 ± 1.68bA	15.69 ± 1.29aCD
OF-25%	20.57 ± 1.75abA	17.94 ± 0.23bcB	16.41 ± 0.66bcB	21.45 ± 0.83aA	16.65 ± 0.49aB
OF-50%	20.80 ± 1.43abA	18.47 ± 0.95bB	16.62 ± 1.06abBC	21.50 ± 1.10aA	16.47 ± 0.56aC
OF-75%	21.02 ± 0.89abA	20.75 ± 0.68aA	17.24 ± 1.03abB	21.85 ± 0.21aA	16.84 ± 0.15aB
OF-100%	21.10 ± 0.46aAB	20.29 ± 0.98aB	17.78 ± 0.48aC	22.04 ± 0.40aA	16.97 ± 0.40aC

Lower-case letters in the same column indicate significant differences in fertilization. Upper-case letters in the same row indicate significant differences in aggregate size (*p* < 0.05). CK, no N fertilizer; CF, N fertilizer without organic fertilizer; OF-25%, OF-50%, OF-75%, and OF-100%, 25%, 50%, 75%, and 100% organic fertilizer.

### Contribution of aggregates to TOC

The contribution to TOC of the > 1 mm aggregate fraction (CAOC) decreased with decreasing particle size and increased with increasing application rates of organic manure (Table 7). In the OF-50%, OF-75%, and OF-100% treatments, CAOC increased in the > 5 mm, 2–5 mm, and 1–2 mm aggregate fractions by 16.27–36.77%, 14.24–37.07%, and 23.50–30.80%, respectively, relative to the CF treatment. In contrast, in the < 1 mm aggregate fractions, CAOC increased with decreasing particle size and decreased with increasing application rates of organic manure. No significant differences occurred between treatments (except compared to CK) in the 0.25–1 mm aggregate fraction, but the OF-50%, OF-75%, and OF-100% treatments had significantly lower CAOC in the < 0.25 mm aggregate fraction than the CF treatment. In general, the macroaggregates (> 0.25 mm) had higher CAOC than the microaggregates (< 0.25 mm).

**Table 7:** Contribution of differently-sized aggregates (%) to total organic carbon in different fertilization treatments in a gravel-mulched field.

Treatments	Aggregates size (mm)				
	> 5	2–5	1–2	0.25–1	< 0.25
CK	16.08 ± 2.15dC	13.67 ± 0.82cC	10.09 ± 0.89dD	22.78 ± 1.81aB	37.34 ± 2.60aA
CF	16.78 ± 1.17dC	14.19 ± 1.47cD	10.81 ± 1.36cdE	21.40 ± 1.03abB	36.76 ± 1.32aA
OF-25%	17.86 ± 0.67cdC	14.82 ± 0.80bcD	11.82 ± 1.02bcE	20.50 ± 1.77abB	35.13 ± 1.89aA
OF-50%	19.51 ± 0.92bcB	16.21 ± 0.83bC	13.35 ± 0.88abD	19.77 ± 1.51bB	31.26 ± 1.80bA
OF-75%	20.95 ± 1.52abB	18.14 ± 0.64aC	13.28 ± 0.11abD	19.42 ± 0.37bBC	28.31 ± 2.53bA
OF-100%	22.95 ± 1.65aA	19.45 ± 1.02aB	14.14 ± 0.24aC	19.10 ± 1.40bB	24.41 ± 1.99cA

Lower-case letters in the same column indicate significant differences in fertilization. Upper-case letters in the same row indicate significant differences in aggregate size (*p* < 0.05). CK, no N fertilizer; CF, N fertilizer without organic fertilizer; OF-25%, OF-50%, OF-75%, and OF-100%, 25%, 50%, 75%, and 100% organic fertilizer.

### TOC storage

TOC storage increased with decreasing particle size in the ≥ 1 mm or < 1 mm aggregate fractions and increased with increasing application rates of organic manure, with the maximum value in the < 0.25 mm fraction (Table 8). However, macroaggregates (> 0.25 mm) had higher TOC storage than microaggregates (< 0.25 mm). In the OF-50%, OF-75%, and OF-100% treatments, TOC storage significantly increased in the macroaggregates (> 0.25 mm), microaggregates (< 0.25 mm), and soil profile (0–20 cm) by 5.74–7.60%, 6.53–8.70%, and 5.91–7.84%, respectively, relative to the CF treatment.

**Table 8:** Total organic carbon storage (Mg per ha) of aggregate fractions in different fertilization treatments in a gravel-mulched field.

Treatments	Aggregates size (mm)						Total storage
	> 5	2–5	1–2	0.25–1	< 0.25	> 0.25	
CK	8.76 ± 0.48bC	9.59 ± 0.17bB	9.83 ± 0.36cAB	9.39 ± 0.18bB	10.33 ± 0.12cA	37.57 ± 1.03b	47.90 ± 1.14b
CF	8.95 ± 0.36bD	9.86 ± 0.21bB	10.13 ± 0.20bcB	9.37 ± 0.18bC	10.57 ± 0.15cA	38.31 ± 0.55b	48.88 ± 0.69b
OF-25%	9.00 ± 0.50bC	9.90 ± 0.30bB	10.42 ± 0.10abAB	9.34 ± 0.30bC	10.80 ± 0.11bcA	38.65 ± 1.01b	49.45 ± 1.12b
OF-50%	9.77 ± 0.28aC	10.31 ± 0.14aB	10.64 ± 0.21aB	9.76 ± 0.16abC	11.26 ± 0.24abA	40.51 ± 0.25a	51.77 ± 0.43a
OF-75%	9.84 ± 0.21aC	10.34 ± 0.15aBC	10.68 ± 0.21aAB	9.89 ± 0.37aC	11.05 ± 0.32abA	40.81 ± 0.91a	51.86 ± 1.18a
OF-100%	10.06 ± 0.20aC	10.43 ± 0.19aBC	10.77 ± 0.06aB	10.05 ± 0.30aC	11.49 ± 0.46aA	41.22 ± 0.62a	52.71 ± 1.02a

Lower-case letters in the same column indicate significant differences in fertilization. Upper-case letters in the same row indicate significant differences in aggregate size (*p* < 0.05). CK, no N fertilizer; CF, N fertilizer without organic fertilizer; OF-25%, OF-50%, OF-75%, and OF-100%, 25%, 50%, 75%, and 100% organic fertilizer.

### Fruit yield of watermelon

**Figure 3:**
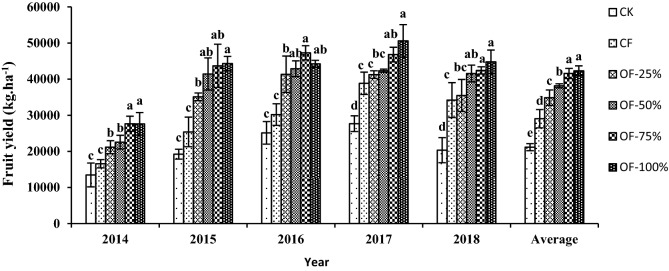
Fruit yield of watermelon over 5 years under different inorganic and organic fertilization treatments in a gravel mulch. Bars within the same year followed by the same letter are not significantly different at *P* = 0.05. CK, no N fertilizer; CF, N fertilizer without organic fertilizer; OF-25%, OF-50%, OF-75%, and OF-100%, 25%, 50%, 75%, and 100% organic fertilizer.

The fruit yield of watermelon increased with increasing application rates of organic manure and was significantly higher in the OF-75% and OF-100% treatments (Fig. 3), which increased by 66.79 and 66.67% in 2014, 74.59 and 72.16% in 2015, 56.64 and 46.49% in 2016, 20.49 and 30.25% in 2017, and 24.02 and 30.94% in 2018, respectively, relative to the CF treatment. Overall, the OF-75% and OF-100% treatments yielded > 40,000 per ha.

## Discussion

### Effects of organic fertilizer on the sizes of soil aggregates

When macroaggregates of soil are stable in water, they reduce surface crusting and help to control soil erosion by water and wind, thus improving infiltration of water and water retention^24,30,39,40^. Obviously, both water infiltration and retention are critical within gravel-mulched fields in the arid loess region of northwestern China. Therefore, the stability of soil aggregates under water influx may be a more sensitive indicator than the properties of bulk soil strength of changes to soil quality and function induced by fertilizer management in the gravel-mulched fields of the region^41^.

Over the 5-year duration of this study, we found that the application of inorganic N fertilizers had no significant effect on the formation and stability of soil aggregates, relative to no N fertilizer. In contrast, the application of organic manure substantially increased the proportion of the macroaggregate fractions (> 1 mm) when compared with soils that received no organic manure, and this is consistent with the results from Ma et al. (2022)^31^, who showed that long-term application of organic fertilizer and the combined application of organic and inorganic fertilizers significantly promote the aggregation of macroscopic aggregates (> 0.25 mm) compared with inorganic fertilizer.

In addition, macroaggregate stability in soil is promoted by the application of organic manure fertilizer. Chen et al. (2020) reported that long-term, excessive application of chemical fertilizer combined with sustained mono-cropping of tobacco reduced water-stable soil aggregate stability, while the use of organic fertilizer increased the proportion of large macroaggregates and MWD and significantly reduced the proportion of microaggregates^28^. The promotion of macroaggregates under an organic fertilizer regime may be related to the accumulation of humic substances, which result from the breakdown of organic matter and increase following the application of organic fertilizer. Tang et al. (2018) reported that soil humic acid (C-HAF), fulvic acid (C-FAF), and humin (C-HUM) increased under the long-term application of a combination of manure and mineral fertilizer^26^. We found that the macroaggregate fractions (R_0.25_) and MWD increased with increasing application rates of organic manure and that the microaggregate fractions decreased. Thus, our results support prior studies indicating the advantage of organic manure fertilizer in macroaggregate formation and, therefore, its capacity to improve soil structure and, consequently, soil function.

### Effects of organic fertilizer on SOC concentrations

Maintaining a high SOC concentration is important, as it directly affects plant productivity by improving soil quality and indirectly affects overall rhizosphere health via impacts on the physical, chemical, and biotic properties of soil^17,42^. However, the correlation of N to SOC remains incompletely understood. Some studies indicate that the application of nitrogen fertilizer can significantly increase the SOC content in soil, and its application rate had a positive correlation with SOC concentration ^25,43^. Moreover, in China, Yang et al. (2003) reported that inorganic-N and NPK fertilization were inadequate for maintaining SOC levels under conventional management of corn with no aboveground crop residues returned to the soil^44^. In contrast, Russell et al. (2005) reported that N fertilization negatively impacted SOC by the density of edaphic bacterial communities^45^. Our study showed that inorganic N applied at rates of 200 kg N per ha (CF treatment) yielded higher SOC contents than the control (CK, no N applied), but the difference was not significant. However, we found that SOC had significant improvement in different-sized aggregate fractions when 50% and more N was replaced with manure than in the CK and CF treatments (Table 4).

Many long-term field experiments have shown that a hybrid fertilization strategy comprising the application of both inorganic and organic fertilizers can increase soil nutrient and SOC concentrations^27,29,46,47^. The increase in SOC concentration under organic fertilization regimes compared to inorganic fertilization probably results from both the direct application of C via organic fertilizer in addition to the effects of organic fertilizer on other soil properties, such as aggregation. In one 18-year study involving the application of organic compost to winter wheat (*Triticum aestivum* L.) and summer maize (*Zea mays* L.), SOC significantly increased by 70.7–121.7% over the initial, pretreatment measurement, while inorganic fertilizer produced an increase in SOC of only 5.4–25.5%, which was not significant over the same timeframe^47^. Similarly, in the rice barley cropping system of eastern China, a 23-year manure application increased the soil SOC content by 25%, while inorganic fertilizer application only increased by 13%^23^. These prior results are highly comparable to ours, in which we found that the application of manure fertilizer yielded overall higher SOC concentrations in differently sized soil aggregates compared to inorganic fertilizer alone, and that the rates of SOC increase were positively correlated with the proportion of organic to inorganic fertilizer applied.

The relationship between soil aggregate sizes and SOC concentrations remains poorly understood. Some previous studies have found that macroaggregates have a higher concentration of SOC than microaggregates possibly because macroaggregates are less likely to fully break down and release C under stress^48^. However, other studies suggest that the stability of SOC is greater in smaller aggregates ^49,50^ because the smaller aggregates represent more of a closed system affording greater physical protection ^51,52^. We propose that the reasons for discrepancies among prior studies may be because that the decomposition of abundant cellulose and sucrose in macroaggregates temporarily leads to an increase in SOC ^53^. Decomposition of cellulose and sucrose in macroaggregates may also occur more quickly due to the favourable biotic (and enzymatic) environment of macroaggregates^47^. Our data are congruent with those of Puget, et al. ^49^ and Ashman, et al. ^50^ in indicating that microaggregates (< 0.25 mm) had the highest TOC content, possibly because newly added C from manure becomes robustly sequestered within the microaggregates.

### Effects of organic fertilizer on soil POC among different aggregate sizes

Permanganate oxidizable carbon (POC) is the active part of SOC that, following processing by microorganisms, can be readily used by plants. Qiu et al. (2015) reported that active carbon (permanganate oxidizable carbon) was the best overall indicator of the soil C status and decreased with increasing cultivation time in gravel-mulched fields^2^. We found that POC and the ratio of POC to TOC were improved under increasing application rates of organic manure. Among differently-sized soil particles, we found that macroaggregates (> 0.25 mm) had higher POC:TOC ratios than microaggregates (< 0.25 mm), which might be attributable to the unfavourable environment for microbially facilitated transitions from inactive to active carbon within the microenvironments of microaggregates^47^. Similar to our results, Tong et al. (2020) also found that the application of organic fertilizer significantly increased soil POC among different-sized soil aggregates^54^.

### Effects of organic fertilizer on TOC storage

The distribution of soil aggregate sizes and their differing organic carbon content (CAOC) objectively reflect the effect of fertilizer management practices on the organic carbon pool of soil. Our results showed that macroaggregates (> 0.25 mm) had higher CAOC than microaggregates (< 0.25 mm), despite microaggregates having higher TOC. As a result, macroaggregates (> 0.25 mm) had greater TOC storage than microaggregates (< 0.25 mm), and approximately 78% of the total SOC was stored in macroaggregates (> 0.25 mm). This finding is consistent with the results of Li et al. (2013), who reported that macroaggregates contained more SOC than microaggregates and that there was a positive relationship between the abundance of macroaggregates and total SOC^55^. Taken together with our results, this finding supports that a greater abundance of macroaggregates facilitates a higher concentration of SOC. Moreover, we found that TOC storage and the CAOC of macroaggregates increased with increasing application rates of organic manure. Similarly, Su, et al. ^56^ determined that approximately 57–64% of the total SOC was stored in macroaggregates (> 0.25 mm) after treatment with organic fertilizer, compared to 54–60% prior to treatment.

### Effect of fertilizer practices on watermelon yield

This study showed that the fruit yield of watermelon increased with increasing application rates of organic manure over 5 years in a gravel-mulched field. We suspect that there are two main reasons for this result:

1. The application of organic fertilizer improved the soil physical structure and chemical properties by increasing soil aggregation and promoting soil stability. The combined application of inorganic and organic fertilizers likely reduced water loss and improved the efficiency of soil hydrological cycling. Improvements in soil hydrology and watermelon yield under organic fertilization regimes were also observed by Du et al. (2020), who reported that organic fertilizer application increased soil water use efficiency by 54.9–176.3%, N-use efficiency in watermelon by 6.9–18.5%, and the watermelon yield by 63.2–156.6% ^57^. Zhou et al. (2013) showed that wheat biomass had a significant correlation with SOC and soil total N in the 0–20 cm soil layer, and mineral fertilizers applied together with cattle manure significantly increased the SOC and total N content in the topsoil layers and dramatically improved winter wheat grain and aboveground biomass yields^58^.
2. The application of organic manure over a 5-year period reduced the typical soil-depletion effects of continuous cropping in a gravel-mulched field. Overall, improvements to the soil quality are likely related to the effects of manure application on the structure and function of the edaphic microbial community ^59^. A balance of fertilization with both organic manure and inorganic NPK may yield changes in the soil microbial composition, especially reducing fungal abundance while increasing bacterial abundance, which promotes the soil cycle and healthy soil structure ^60^.

In general, our results support the incorporation of both inorganic and organic fertilizers as part of an integrative soil fertility management strategy for improving the watermelon yield.

## Conclusions

The partial replacement of chemical fertilizer with organic manure significantly improved the proportion of macroaggregates as well as the SOC content of different -sized aggregates in the topsoil and, consequently, increased watermelon yields relative to chemical fertilizer alone. Long-term application of inorganic fertilizers is inadequate for maintaining SOC and soil nutrient levels, especially under conventional management strategies of gravel-mulched fields. In contrast, the partial or complete replacement of chemical fertilizer with organic manure may recover and maintain soil quality, thus overcoming this constraint on the utilization of gravel-mulched fields. Our work has implications for the sustainable development of the watermelon industry and protection of the natural and agroecological environment in the Loess Plateau of arid northwestern China.

## Supplementary Information

Supplementary Information.

## Data Availability

All data generated or analyzed during this study are included in this published article and its Supplementary Information files.
